# Perineural Invasion in Pancreatic Ductal Adenocarcinoma: Recapitulating Its Importance and Defining Future Directions

**DOI:** 10.1002/ueg2.70118

**Published:** 2025-09-22

**Authors:** Martin Lovecek, Esra Dirimtekin, Ingrid Garajová, Giulia Gasparini, Stefano Crippa, Elisa Giovannetti, Dana Sochorova, Carmen Mota Reyes, Ihsan Ekin Demir, Pinar Uysal‐Onganer, TRANSPAN PNI Group

**Affiliations:** ^1^ Department of Surgery, University Hospital Olomouc Fakultni nemocnice Olomouc Olomouc Czech Republic; ^2^ Cancer Mechanisms and Biomarkers Research Group University of Westminster London UK; ^3^ Medical Oncology Unit Azienda Ospedaliero‐Universitaria di Parma Parma Italy; ^4^ Division of Pancreatic Surgery, Pancreas Translational & Clinical Research Center Universita Vita Salute San Raffaele Milan Italy; ^5^ Department of Medical Oncology, Lab of Medical Oncology, Cancer Center Amsterdam Amsterdam UMC Locatie VUmc Afdeling Medische Oncologie Amsterdam the Netherlands; ^6^ Cancer Pharmacology Lab, AIRC Start‐Up Unit Fondazione Pisana per la Scienza San Giuliano Terme Italy; ^7^ Department of Surgery Tomas Bata Hospital Zlin Czech Republic; ^8^ Department of Surgery, TUM University Hospital Klinikum rechts der Isar der Technischen Universitat Munchen Krankenhausapotheke Munich Germany; ^9^ Else Kröner Clinician Scientist Professor for Translational Pancreatic Surgery Munich Germany; ^10^ Cancer Mechanisms and Biomarkers Research Group University of Westminster London UK

**Keywords:** biomarkers, pancreatic cancer, pancreatic ductal adenocarcinoma, perineural invasion, prognostic marker, therapeutic strategies, tumor aggressiveness

## Abstract

Pancreatic ductal adenocarcinoma (PDAC) remains one of the most lethal malignancies, mainly due to its aggressive nature, early metastasis, diagnosis at late stages, and limited response to systemic anticancer therapy. Perineural invasion (PNI), defined as the infiltration of neoplastic cells along nerve fibers and within nerve sheaths, is emerging as a critical determinant of PDAC aggressiveness. This position paper by the *TRANSPAN PNI Group on Perineural Invasion* synthesizes current evidence on the molecular features and clinical implications of PNI in PDAC, compares its prognostic significance in other malignancies, and describes novel biomarker strategies including potential therapeutic interventions.

## 1 Introduction

Perineural invasion (PNI) refers to the infiltration and spread of tumor cells along nerve sheaths, including the epineural, perineural, and endoneurial spaces, particularly frequent in pancreatic ductal adenocarcinoma (PDAC). It is increasingly recognized as a distinct route of cancer dissemination, separate from vascular and lymphatic invasion, and is associated with poor prognosis across various malignancies, including prostate, cervical, gastrointestinal, and head and neck cancers [1]. In neurotropic tumors like PDAC, malignant cells can travel along nerves beyond primary lesions, limiting surgical resection and contributing to recurrence or incomplete removal. Histopathological assessment of PNI is currently binary (present/absent), but emerging classifications, distinguishing intrapancreatic intratumoral, peritumoral, and extrapancreatic PNI, along with severity scoring based on nerve size and invasion extent, offer a more nuanced evaluation [2], representing early steps toward standardization, though further prospective validation is required. Despite its near‐universal presence PDAC, perineural invasion (PNI) remains inconsistently classified, underreported, and poorly integrated into clinical decision‐making.

The aim of this position paper is to capture real‐world insights into the recognition, classification, and perceived clinical relevance of PNI, and to identify barriers to its standardized assessment and integration into prognostic or therapeutic pathways. This work represents a foundational step toward building consensus and guiding future standardization efforts in PNI reporting and management.

## 2 Perineural Invasion in PDAC: The Minimum You Should Know

**FIGURE 1:**
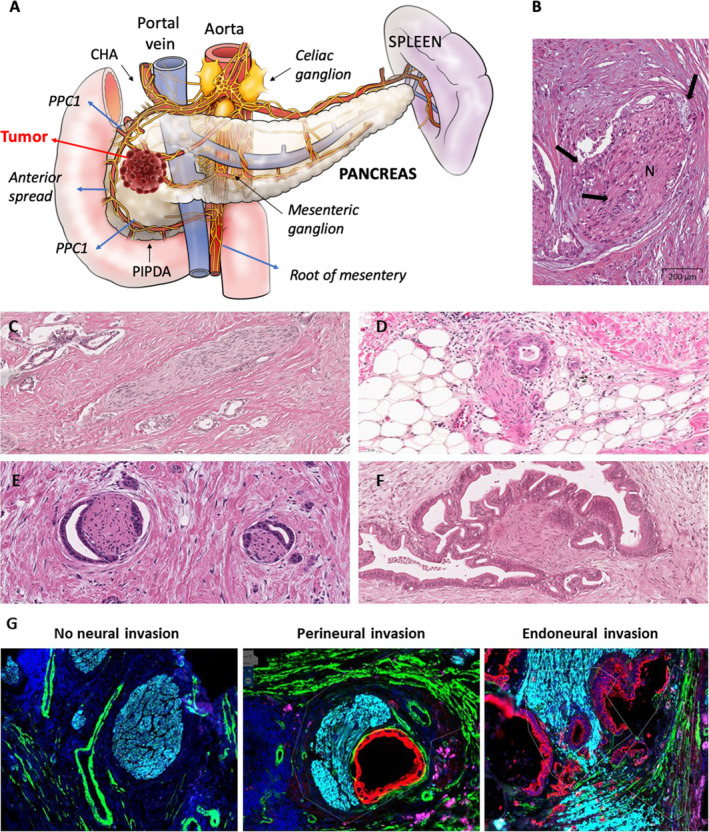
(A) Anatomy of the pancreas and major routes of perineural spread. The four major routes of perineural spread (Anterior, Root of Mesentery, and Plexus Pancreaticus Capitalis) are illustrated. [4]. (B) Histological evidence of perineural invasion in a patient with PDAC: Neoplastic glands (arrows) surround a nerve fiber (N), hematoxylin and eosin (H&E) stain. (C) Representative H&E stained human PDAC slide with a nerve exhibiting no PNI. (D) Representative H&E stained human PDAC section showing a nerve with short distance perineural contact with cancer cells (mild PNI). (E) Genuine perineural invasion/PNI with a semilunar arrangement of cancer cells around two intrapancreatic nerves. (F) Endoneural invasion with destruction of the nerve structure. (G) Multiplex immunofluorescence (IF) images of nerves without and with nerve invasion. Nerves in blue, cancer cells in red, and aSMA + myofibroblasts or vessel walls in green. CHA = common hepatic artery, PPC1 and PPC2 = plexus pancreaticus capitalis, PIPDA = posterior inferior pancreaticoduodenal artery.

PDAC exhibits PNI in nearly 100% of cases, far exceeding other solid tumors (Table 1) [3]. The prominence and severity of PNI in PDAC likely reflect the pancreas' unique anatomical position and its rich surrounding nerve plexus (Figure 1).

**TABLE 1:** PNI comparison among GI cancers.

Malignancy	PNI prevalence	Prognostic impact	PNI characteristics
Pancreatic cancer	Extremely high (60%–100% in histological studies)	Strongly linked to poor prognosis, early recurrence, and reduced survival.	Dense neural network facilitates aggressive PNI. Robust tumor‐nerve interactions with severe pain syndromes.
Cholangiocarcinoma	High (40%–80%, especially in perihilar tumors)	Associated with worse outcomes, higher recurrence, and shorter survival.	Similar to PDAC in its tropism for nerve plexuses, especially near bile ducts.
Colorectal cancer	Moderate (20%–40%)	Associated with advanced stage, poor prognosis, and recurrence risk.	Neural invasion is less prominent; rarely leads to extensive nerve‐associated symptoms.
Gastric cancer	Moderate (15%–50%, depending on stage)	Correlates with advanced stage and shorter survival.	Tumor tropism for nerves is less aggressive than PDAC.
Esophageal cancer	Moderate to high (30%–60%)	Related to local invasion, poor prognosis, and increased recurrence	PNI is often localized, unlike the extensive nerve plexus involvement seen in PDAC.
Hepatocellular carcinoma (HCC)	Rare (< 10%)	Rare occurrence makes significance unclear.	Lacks extensive nerve invasion pathways.

The interaction between pancreatic tumor cells and nerves is driven by a dynamic molecular network within the tumor microenvironment. For example, hypoglycaemia‐induced upregulation of neurotrophins such as nerve growth factor (NGF) has been linked to reduced survival in diabetic patients [5]. NGF activates TrkA receptors, promoting angiogenesis, epithelial‐mesenchymal transition (EMT), and tumor invasiveness [6], although its role can be context‐dependent, acting as either pro‐oncogenic or pro‐apoptotic. Increased neural density and hypertrophy correlate with pain severity in PDAC patients [7].

PNI is considered an early event in PDAC progression and is associated with poor outcomes, even in early‐stage, node‐negative cases. Neurotropism has been observed not only in invasive PDAC but also in precursor lesions, such as pancreatic intraepithelial neoplasia, in both murine and *vitro* models, suggesting that neural invasion contributes to early tumor evolution [8]. Although some studies link PNI to local recurrence, potentially due to tumor spread via intrapancreatic nerves [9], others found no such association [10], warranting prospective investigation. Patients with high neural invasion scores may represent a distinct subgroup that could benefit from tailored postoperative therapies.

The impact of neoadjuvant chemoradiation therapy on PNI remains inconclusive. While some studies report a reduction in PNI frequency following neoadjuvant treatment, for instance, from 95.4% to 72.5% [11],and from 88% to 70% [12], others have observed only a non‐significant trend toward reduced PNI incidence [10]. These discrepancies raise concerns about the consistency and robustness of the observed therapeutic effect. Variability in neoadjuvant treatment protocols and patient population heterogeneity likely contribute to the conflicting findings. Furthermore, a more precise and standardized definition of PNI is necessary to accurately evaluate the impact of neoadjuvant therapy. Notably, in advanced‐stage disease, the evaluation of PNI is limited by the absence of surgical specimens. Nonetheless, PNI remains a critical biological feature and warrants continued investigation as a potential prognostic and therapeutic target.

## 3 Real‐World Perception of PDAC Across Clinicians: Survey Results

To evaluate the current clinical value of PNI in PDAC across clinicians, we designed a cross‐sectional survey that aimed to explore the perceived clinical significance of PNI in PDAC among pancreatologists. The goal was to understand current practices, awareness, and interdisciplinary perspectives on PNI classification across national and university hospitals.

The survey was developed through an iterative process. Initially, a preliminary set of questions was developed focused on globally assessing the perception of the role of PNI among pancreatologists. The initial draft questionnaire underwent pilot testing and was refined by the multidisciplinary TRANSPAN PNI group. The final survey, featuring concise and structured questions, was then administered electronically. We reached out to a broad group of pancreatologists, specifically targeting members of the European Pancreatic Club. We kindly requested participation from colleagues who regularly deal with PNI in PDAC as part of their daily practice. Respondents were asked to reflect on their clinical reality and institutional protocols, ensuring responses represented the standard of care across key disciplines involved in PDAC diagnosis and management. While efforts were made to include various institution types, academic centers might be overrepresented compared to community hospitals, potentially reflecting differences in resources and caseload (Table 2).

**TABLE 2:** Survey overview on the recognition and clinical relevance of PNI in PDAC.

PNI survey	Yes	No
Pathologist		
Do you identify PNI in resected PDAC specimens?	17/24 (%71)	7/24 (%29)
Do you consider PNI in PDAC to be an important prognostic factor/aggressiveness factor of PDAC?	14/24 (%58)	10/24 (%42)
Do you know any PNI classification system?	6/24 (%25)	18/24 (%75)
Are you able to implement a PNI classification system in your clinical practice?	14/24 (%58)	10/24 (%42)
Oncologist		
Do you consider PNI in PDAC to be an important prognostic factor/aggressiveness factor of PDAC?	16/24 (%66)	8/24 (%34)
Do you consider PNI to be a factor affecting type of adjuvant therapy in radically resected PDAC patients?	3/24 (%12)	21/24 (%88)
Do you know any PNI classification system?	10/24 (%41)	14/24 (%59)
Do you think it might be important to use a PNI classification system to know more about PNI in our PDAC patients?	12/24 (%50)	12/24 (%50)
Surgeon		
Do you consider PNI in PDAC to be an important prognostic factor/aggressiveness factor of PDAC?	36/41 (%87)	5/41 (%13)
Do you consider PNI to be a factor affecting loco regional recurrence of PDAC?	34/41 (%82)	7/41 (%18)
Do you perform mesopancreas resection during pancreatectomy routinely?	25/41 (%60)	16/41 (%40)
Do you evaluate locoregional recurrence following pancreatectomy during follow up?	29/41 (%70)	12/41 (%30)

*Note:* This table presents the results of a survey conducted among pathologists (*n* = 24), oncologists (*n* = 24), and surgeons (*n* = 41) from the Czech Republic, Germany, the United Kingdom, and Turkiye. It demonstrates that the majority of pathologists identified PNI in resected PDAC specimens, oncologists recognized its clinical significance, and surgeons unanimously considered PNI a critical prognostic marker for tumor aggressiveness.

## 4 Molecular Mechanisms and Biomarker Profiling of PNI

Emerging biomarkers such as microRNAs (miRNAs), circulating tumor cells (CTCs), long non‐coding RNAs (lncRNAs), exosomal cargo, and DNA fragmentation have been implicated in PNI. Advances in genetic and epigenetic profiling, enhanced by artificial intelligence (AI)‐driven analytics, are deepening our understanding of PNI mechanisms and paving the way for novel diagnostic and therapeutic strategies.

*Aberrant miRNA expression* is frequently observed in malignancies, including PDAC [13, 14]. In the context of PNI, elevated levels of miR‐10b and miR‐196a are associated with increased neural invasion and poor prognosis, whereas tumor‐suppressive miRNAs such as miR‐34a and miR‐200c inhibit EMT and neural infiltration.

*CTCs* expressing neural adhesion molecules such as L1CAM suggest a potential link between systemic dissemination and PNI [8]. Single‐cell analyses have revealed subpopulations of CTCs with neural invasive properties, particularly those expressing EMT markers and neurotrophic factors. Liquid biopsies enable real‐time monitoring of these CTC dynamics, offering a non‐invasive means to assess disease progression and therapeutic response.

*LncRNAs,* such as MALAT1, HOTAIR, and H19, have been implicated in promoting PNI by modulating EMT‐related genes and the tumor microenvironment [15]. Their dysregulation highlights their potential as biomarkers and therapeutic targets. Emerging approaches, including antisense oligonucleotides and CRISPR‐based technologies, offer promising strategies to inhibit lncRNA‐driven neural invasion.

*Exosomes*, as mediators of tumor–nerve communication, carry oncogenic proteins, lipids, and nucleic acids that may enhance PNI. Reciprocal exosome uptake between tumor and neural cells may amplify neural invasion and metastasis. However, there is limited knowledge on the role of exosomes in the nerve‐cancer crosstalk. A recent study showed that neuro‐invasive cancer cells secrete exosomes that contain the low‐affinity NGF receptor p75NTR and that these exosomes are enriched in the plasma of PDAC patients with high severity of PNI and worse prognosis. Cancer cell‐derived, p75NTR‐containing exosomes led to dedifferentiation and increased carcinotropic migration of Schwann cells [16].

*DNA fragmentation,* associated with genomic instability and resistance to apoptosis, has been proposed as a PNI marker. Elevated levels of circulating fragmented DNA and tumor‐specific mutations in cell‐free DNA (cfDNA) correlate with poor clinical outcomes and aggressive tumor behavior [17]. Additionally, aberrant DNA methylation and mutations in genes such as KRAS and TP53 are linked to PNI [15].

*AI* is transforming cancer research by uncovering patterns in complex genomic and histopathological datasets. AI models can predict PNI presence and progression with high accuracy [18], supporting early detection and personalized therapeutic strategies through advanced epigenetic and genetic profiling. Table 3 summarizes the current models that are in use to study PNI in PDAC.

**TABLE 3:** Experimental models for investigating PNI in PDAC.

	Model	Key features	Applications	Limitations
In vitro	Co‐culture systems	Cancer cell lines co‐cultured with dorsal root ganglion (DRG) neurons	Direct cell‐cell interactions (cancer neurotropism and neurite outgrowth); paracrine crosstalk	Lacks 3D structure
	Transwell assays; microfluidic devices	Chambers simulating nerve‐cancer microenvironments	Chemotaxis of neural‐secreted cytokines on cancer cells; high control over physical and chemical gradients and real‐time imaging	Expensive setup: technical expertise required
	Organotypic nerve‐tumor co‐cultures	DRG explants co‐cultured with tumor spheroids/organoids	3D representation of tumor‐nerve interactions and signaling	Limited scalability; may not capture all systemic interactions
In vivo	Orthotopic nerve invasion models	Cancer cells injected directly into the sciatic nerve	Nerve invasion, tumor growth and nerve function monitoring	Somatic nerves instead of autonomic nerves typically found in PDAC; injury induced by injection precludes studies of nerve‐damage response to cancer invasion
	Orthotopic xenograft models	Cancer cells injected into the murine pancreas	Nerve‐cancer crosstalk; cancer migration; PNI incidence recorded; test therapeutic interventions	Lack of human‐like neuropathic changes
	Genetically engineered mouse models (GEMMs)	*TPAC* mouse model [19] (*Ela1‐Tgf‐α; p48‐Cre; trp53lox/lox; RelAlox/lox)*, first to consistently demonstrate human‐like PNI	Molecular mechanisms underlying nerve‐cancer interactions, test therapeutic interventions	Expensive and time‐consuming
	Patient‐derived orthotopic xenografts (PDXs)	Patient‐derived PDAC as primary or passaged cancer cells or organoids	Genomic/transcriptomic analyses; metabolism; test (personalized) therapeutic interventions	Expensive; requires human samples and immunocompromised mice

## 5 Therapeutic Strategies Targeting PNI

PNI contributes significantly to the decline in quality of life in PDAC patients due to tumor progression and neuropathic pain, making it a compelling therapeutic target. While anti‐neurogenic therapies have traditionally been utilized for managing neurological disorders and pain syndromes, their application in oncology to mitigate nerve infiltration remains underexplored. Recent research has focused on targeting various molecular and cellular mechanisms underlying PNI, offering potential therapeutic strategies that may improve patient outcomes.

**FIGURE 2:**
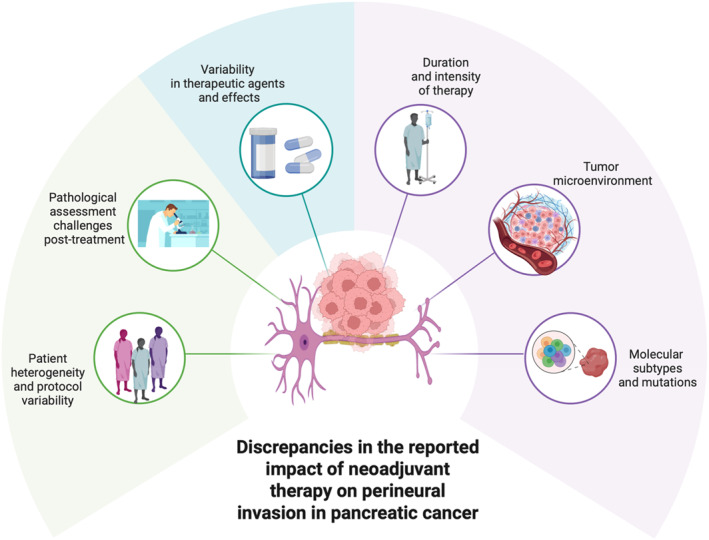
Potential reasons for the discrepancies in the reported impact of neoadjuvant therapy (NAT) on PNI in PDAC extend beyond patient heterogeneity and protocol variability. The heterogeneity of PDAC at the molecular and morphological level in the cancer cells and in the tumor microenvironment, the various therapy regimens, and the different approaches in the histopathological assessment account for the reasons behind different PNI severities after neoadjuvant therapy. Figure was created by BioRender.

However, even after effective targeting of PNI in the clinical setting, comparing the outcomes of different studies remains challenging due to tumor heterogeneity and inconsistencies in the reporting of PNI (Figure 2). Factors that may contribute to these inconsistencies include: (A) *pathological assessment challenges post‐treatment*, because of (1) therapy‐induced tissue changes: NAT can lead to fibrosis, necrosis, and inflammation, complicating the identification of viable tumor cells and PNI [20], (2) underestimation of residual disease: Pathologists may miss microscopic foci of PNI in areas of dense fibrotic tissue post‐treatment, prompting new studies using artificial intelligence [21], and (3) inter‐observer variability: There is no universally standardized method for assessing PNI, and subjective differences in interpretation across institutions or individuals can lead to inconsistent reporting; (B) *variability in therapeutic agents and effects* because of (1) chemotherapy regimens: A meta‐analysis indicated that neoadjuvant FOLFIRINOX was associated with a lower incidence of PNI compared to gemcitabine plus nab‐paclitaxel (70.5% vs. 79.8%), suggesting differential efficacy in reducing PNI [22]. When combined with chemotherapy (chemoradiation), radiation may have a direct impact on perineural tissues and reduce PNI, but results might vary based on dose and targeting and/or (2) neurotropic effects: Certain agents may influence tumor‐neural interactions differently, potentially affecting PNI rates. For example, agents that interfere with the NGF or CXCR4 pathways could impact perineural spread. In addition, targeting the CXCL12/CXCR4 axis with specific antagonists offers a promising strategy to mitigate PNI and overcome chemoresistance in PDAC; (C) *duration and intensity of therapy* (i.e., dose intensity and completion rates, i.e. variations in how much of the planned therapy is completed (due to toxicity or patient tolerance), can lead to inconsistent therapeutic effects on PNI); (D) *tumor microenvironment*, including (1) stromal remodeling: NAT alters the pancreatic tumor microenvironment, including the desmoplastic stroma, which may influence PNI detection and its biological significance, and (2) immune modulation: Some regimens might affect immune cell infiltration and cytokine profiles, which could either suppress or promote perineural invasion depending on the context; and (E) *molecular subtypes and mutations*, such as (1) heterogeneity of PDAC subtypes: Tumors with basal‐like or quasi‐mesenchymal phenotypes may exhibit higher neurotropism, potentially overriding therapeutic effects and (2) type of *mutations*: Specific mutations could influence the propensity for PNI and the response to NAT. For instance, loss of SMAD4 function is linked to increased metastatic potential and may influence PNI. However, SMAD4 inactivation has also been associated with resistance to radiotherapy, potentially due to the promotion of autophagy and reactive oxygen species production, which help tumor cells survive treatment [23].

### 5.1 Targeting Chemokines

Chemokine signaling plays an essential role in tumor‐nerve interactions, with specific chemokine axes contributing to PNI and tumor progression [24]. The CXCL12/CXCR4 axis is particularly significant in PDAC [25],as CXCR4 is overexpressed in cancer cells while its ligand, CXCL12, is upregulated in dorsal root ganglia (DRG). Inhibition of this axis with AMD3100 has demonstrated efficacy in preclinical models, reducing metastatic potential and nerve infiltration. Similarly, the CCL21/CXCL10 and CCL2 axis has been identified as a key mediator of neural remodeling, PNI, and cancer‐associated pain in PDAC [24]. Anti‐CCL2 antibodies have shown promise in colorectal cancer models by inhibiting both neural invasion and angiogenesis through suppression of Akt and MAPK pathways [26]. Furthermore, CX3CL1/CX3CR1 overexpression has been linked to PNI severity in PDAC, highlighting another potential therapeutic target for disrupting neural dissemination as well as PDAC cell viability [27]. In a recently reported, human‐like neuro‐invasive mouse model of PNI termed PDAC, Wang et al. showed that CCL2 from DRG neurons chemoattracts cancer cells to nerves over the CCR4 receptors. Pharmacological inhibition of CCR4 with specific inhibitors markedly reduced PNI in this novel model [19].

### 5.2 Targeting Axon‐Guidance Factors

Axon‐guidance molecules, including semaphorins (SEMAs) and Slit glycoproteins, play crucial roles in neural development and cancer progression. In PDAC, SEMA3D and its receptor plexin D1 (PLXND1) have been implicated in tumor invasion and nerve infiltration [28]. Early‐phase clinical trials are evaluating the safety of anti‐SEMA4D antibodies, such as pepinemab, in solid tumors, including PDAC [29], offering a novel approach to targeting neural invasion. Slit proteins and their Robo receptors also influence PNI and tumor dissemination [30]. Slit glycoprotein‐2 has also demonstrated inhibitory effects on metastasis and neural infiltration in PDAC models [31]. These findings suggest that axon‐guidance molecules could serve as valuable therapeutic targets in preventing PNI and reducing tumor progression.

### 5.3 Targeting Neurotransmitters/‐Peptides

Several pharmacological agents modulating the autonomic nervous system have shown potential in cancer therapy. Beta‐blockers, such as propranolol, bupranolol and metoprolol, have been investigated for their ability to reduce NGF expression, nerve density, and tumor progression in PDAC models [32]. The combination of beta‐blockers with chemotherapy has demonstrated improved survival outcomes in preclinical studies [33]. Targeting the parasympathetic nervous system has also yielded promising results. Blocking the muscarinic receptor CHRM3 with darifenacin inhibited prostate cancer growth in vitro and in vivo [34]. Interestingly, CHRM3 overexpression has been associated with resistance to gemcitabine in PDAC, suggesting that parasympathetic modulation could influence chemotherapy responsiveness [35]. Additionally, neuropeptide‐based therapies have emerged as potential strategies for cancer‐associated pain relief. Resiniferatoxin, a TRPV1 agonist, has been shown to induce apoptosis in pancreatic cancer cells while alleviating pain, as recently reviewed [36]. CGRP antagonists, including erenumab, galcanezumab, fremanezumab, and gepants, have demonstrated efficacy in migraine treatment and may hold promise for cancer pain management [37]. Lastly, the substance P (SP)/neurokinin‐1 receptor (NK‐1R) system has also been implicated in pancreatic cancer progression [38]. NK‐1 R antagonists, such as aprepitant, are already used in oncology as antiemetics and have shown potential in inhibiting PDAC cell proliferation. Stratifying patients based on TACR1 expression levels may enhance the effectiveness of NK‐1R‐targeted therapies [39].

### 5.4 Targeting the NGF‐TrkA Signaling Pathway and Other Neurotrophins

NGF and its receptor TrkA play critical roles in neural development and pain signaling. Mutations affecting TrkA or NGF result in congenital insensitivity to pain, highlighting their involvement in nociceptive pathways. NGF‐TrkA signaling axis facilitates nerve infiltration and contributes to cancer‐associated pain, making it a viable target for therapeutic intervention [24]. Various approaches to blocking this pathway have been investigated, including NGF sequestration, inhibition of NGF‐TrkA binding, direct TrkA inhibition, and disruption of downstream signaling cascades, as recently reviewed [40]. Moreover, larotrectinib, a pan‐Trk inhibitor, has been effective in reducing cancer‐associated pain and modifying sensory nerve remodeling in sarcoma models [41].

Clinical trials have evaluated NGF‐blocking antibodies such as tanezumab, fasinumab, and fulranumab for chronic pain conditions [42]. Despite their efficacy in pain relief, adverse effects, including osteonecrosis and autonomic nervous system disturbances [43], have hindered their progression into oncology. Future studies may focus on optimizing these therapies to balance efficacy and safety in cancer treatment.

### 5.5 Targeting Adhesion Factors and Proteins

**FIGURE 3:**
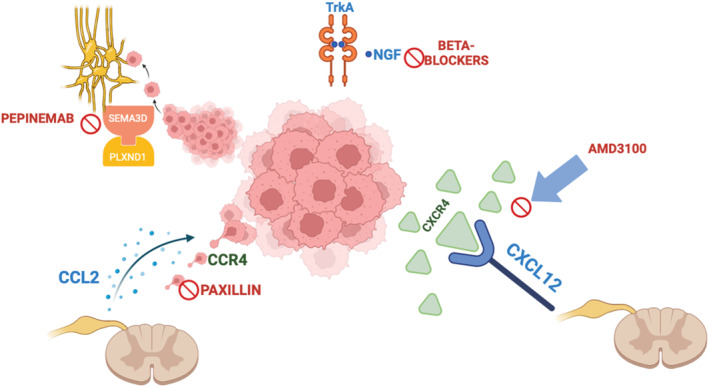
Schematic representation of tumor–nerve crosstalk in the perineural niche. This figure illustrates key molecular pathways mediating bidirectional communication between tumor cells and nerves, including neurotrophic factors, adhesion molecules, and inflammatory mediators. Figure was created by BioRender.

Cell adhesion molecules play a pivotal role in PNI by facilitating tumor cell interactions with surrounding nerves. L1 cell adhesion molecule (L1CAM), secreted by Schwann cells, acts as a potent chemoattractant for cancer cells, promoting neural invasion via the activation of MAP kinase signaling and upregulation of matrix metalloproteinases (MMP‐2 and MMP‐9) [44]. Conversely, L1CAM inhibition through monoclonal antibodies significantly reduces PNI in vivo. Moreover, a recent study observed that TGF‐β1 secreted by pancreatic stellate cells negatively regulated L1CAM expression, through canonical TGF‐β‐Smad2/3 signaling, leading to a more aggressive PDAC phenotype. However, inactivation of TGF‐β1 signaling in PSCs strongly reduced the aggressiveness of PDAC cells [45]. Silencing MMP1 mitigated EMT and Schwann‐like differentiation and led to suppression of PNI in PDAC [46]. Furthermore, CLDN4, has been implicated in neural invasion [47]. Low CLDN4 expression correlates with aggressive disease and increased nerve infiltration, whereas its overexpression is linked to improved survival [48]. These findings highlight the potential of targeting adhesion molecules and proteases in therapeutic strategies aimed at mitigating PNI (Figure 3).

## 6 Neuroscience Integration and Novel Interventions

As PNI is a recognized pathway for cancer dissemination, emerging evidence indicates that cancer cells hijack embryonic developmental programs to access nerves. Specifically, TGFβ1 secreted by tumor cells induces EMT in perineural epithelial cells, promoting their migration and secretion of MMPs, which degrade the perineural matrix and facilitate nerve infiltration [48]. Beyond structural exploitation, functional interactions between neurons and cancer cells in PNI remain underexplored. Our recent unpublished findings (under revision) revealed synaptic connections between pancreatic cancer cells and axons within invaded nerves, suggesting active bidirectional communication. These insights call for deeper investigation into the functional dynamics of nerve–tumor interactions during PNI. We propose the following research directions:

1. *Subtyping of nerve fibers and their neurotransmitters in PNI across different cancers*: In pancreatic cancer, PNI was found to be more prevalent in nerves that exhibit a loss of sympathetic (TH+) and cholinergic (ChAT) [49]. Whether such suppression of autonomic fibers in PNI of other cancers is similarly present, should be further investigated and can provide insights into the functional implications of PNI in organ (dys‐)function in cancer.
2. *Impact of PNI on peripheral glia activity*: PNI seems to be associated with activated glia in pancreatic cancer, both in the periphery and the CNS [50]. It is yet unknown how PNI affects the prime function of peripheral glia, that is myelination. As an effect of myelination would theoretically affect the signal transduction efficacy, such a hitherto unknown effect may have key implications for neural influence on organ function and on cancer growth.
3. *Characterization of synaptic activity between cancer cells and axons*: The discovery of peripheral cancer‐neuron synapses implies fast short‐distance interaction between cancer cells and neurons in the periphery. This observation raises several exciting questions, for example, on the type of presynaptic neurotransmitters and post‐synaptic receptors that mediate these interactions, on the extent of co‐activated cancer‐neuron networks, and on the nature of the putative electrical activity of nerve‐invading cancer cells. The presence of such synaptic activity and network activity can be tracked by employing neuroscience tools such as voltage‐sensitive dye imaging, genetically encoded calcium indicators, and tissue slice cultures of peripheral cancers.

As such, studying PNI through integrating these neuroscientific aspects will likely widen our horizon in relation to cancer neuroscience and may open previously unconsidered therapeutic windows.

## 7 Emerging Classification and Severity Scoring Systems: Criteria, Challenges, and Future Directions

Efforts to classify and score PNI severity have evolved significantly, as demonstrated by the proposal of severity scoring systems by independent groups. The common idea is that a more detailed assessment of PNI would result in improved stratification of patient outcomes.

However, current classification systems for PNI all rely on the assessment of PNI on routine histology specimens. As such, they fall short of several aspects, as listed below:

1. UICC staging only reports the presence of PNI versus its absence. Although the presence of PNI is prognostic, several studies have convincingly shown the relevance of PNI severity [2, 10]. As such, for improved stratification, severity assessment is necessary
2. Standardized assessment of the severity of PNI is challenging. While some studies differentiated between the presence of intra‐versus perineural contact between cancer cells and nerves [3], others introduced other morphological parameters such as the size of invaded nerves (< vs. > 3 mm). It is not yet clear which of these different morphological aspects matter more than the others regarding prognostication.
3. Precise assessment of PNI necessitates evaluation of all nerves on a section, which can only be reliably achieved through immunohistochemistry. However, in routine clinical practice, double immunostaining for nerves and cancer cells is not performed in PDAC specimens.
4. In our view, the near‐perfect assessment of PNI severity in PDAC should involve: 3D reconstruction of serial sections of PDAC immunostained for both neural and cancer cell markers, accompanied by automated digital recognition of nerves and automated deep learning‐based assessment of PNI. Such efforts combining 3D reconstruction with deep learning‐based automated PNI assessment strategies are currently being explored in the research groups of the authors and are thus underway.

In our view, the near‐perfect assessment of PNI severity in PDAC should involve 3D reconstruction of serial sections of PDAC immunostained for both neural and cancer cell markers, accompanied by automated digital recognition of nerves and automated deep learning‐based assessment of PNI. Such efforts combining 3D reconstruction with deep learning‐based automated PNI assessment strategies are currently being explored in the research groups of the authors and are thus underway.

To overcome these limitations, the most pressing challenges are as follows:

- *Validation*: Current PNI scoring systems require rigorous validation in diverse external patient cohorts.
- *Differentiation Levels*: Scoring systems vary, with some distinguishing merely between invasion and no invasion, while others introduce intermediate stages (e.g., epineural association).
- *Nerve Size Consideration*: Despite evidence linking nerve hypertrophy with invasion severity, most scoring systems focus on nerve quantity rather than nerve dimensions.
- *Implementation Challenges*: Manual scoring is labor‐intensive, highlighting the necessity for machine learning algorithms capable of automated recognition of PNI patterns, currently under development in the laboratories of the principal investigators of the TRANSPAN PNI group.

Addressing these challenges and refining scoring methodologies will significantly enhance clinical applicability and prognostic accuracy in managing cancer‐related PNI.

## 8 Current Clinical Trials Targeting PNI

Despite its clinical significance, there is still a major lack of trials that specifically target PNI. Due to its independent prognostic importance in PDAC, it is, however, likely that reducing the severity of PNI might also exert a beneficial influence on the overall tumor spread and the cause of the disease. Furthermore, we believe that in resected PDAC, PNI severity can, due to its prognostic role, be used to stratify patients into high‐versus low‐risk groups for more aggressive adjuvant therapy, for example, chemoradiotherapy as opposed to chemotherapy only.

Below in Table 4, we summarized the clinical trials that considered PNI in PDAC in their design (derived from ClinicalTrials.gov):

**TABLE 4:** Current clinical trials that included PNI in their study design.

NCT Number	Study title	Study URL	Study status	Sponsor/host institution	Study type
NCT04024358	A new scoring system for perineural and vascular invasion in pancreatic cancer	https://clinicaltrials.gov/study/NCT04024358	ACTIVE_NOT_RECRUITING	Massimo Falconi	Observational
NCT06747481	Neutrophil‐to‐lymphocyte ratio and CA 19.9 as predictors of perineural invasion in pancreatic cancer	https://clinicaltrials.gov/study/NCT06747481	RECRUITING	University of Las Palmas de Gran Canaria	Observational
NCT06616688	Innovative therapeutic treatments to inhibit perineural invasion in pancreatic adenocarcinoma	https://clinicaltrials.gov/study/NCT06616688	NOT_YET_RECRUITING	IRCCS San Raffaele	Observational
NCT03042442	Relation between cachexia, diabetes and periNeural invasion in PANcreatic cancer‐ biomarkers Substudy	https://clinicaltrials.gov/study/NCT03042442	COMPLETED	Iuliu Hatieganu university of medicine and pharmacy	Observational
NCT01129167	Frequency of methods of local invasion of pancreatic adenocarcinoma	https://clinicaltrials.gov/study/NCT01129167	COMPLETED	Columbia university	Observational
NCT04087889	Individual patient expanded access IND of Hope biosciences first blood Relative allogeneic adipose‐derived mesenchymal Stem cells (HB‐adMSCs) for pancreatic cancer	https://clinicaltrials.gov/study/NCT04087889	NO_LONGER_AVAILABLE	Hope biosciences	Expanded access
NCT04400357	Robotic versus open pancreaticoduodenectomy for pancreatic and periampullary tumors	https://clinicaltrials.gov/study/NCT04400357	UNKNOWN	Ruijin hospital	Interventional

## 9 Conclusions and Future Perspectives

PNI is a key driver of PDAC aggressiveness, recurrence, and morbidity, yet remains underexplored. Advances in histopathological classification, molecular profiling, and AI‐driven analysis offer new avenues to elucidate the mechanisms of PNI. Targeting chemokines, neurotrophins, adhesion molecules, and neural synapses presents promising therapeutic potential. To accelerate translational impact, development of human‐relevant models, including organoids, 3D co‐culture systems, organ‐on‐a‐chip platforms, and human‐like animal models, is essential for dissecting tumor–nerve interactions. Integrating these with AI‐guided drug discovery and precision medicine approaches will enhance target identification and therapeutic efficacy. Additionally, combining nerve‐targeting strategies with chemo‐ and immunotherapy may unlock synergistic treatment effects.

A deeper mechanistic understanding of PNI will be critical for developing effective personalized therapies for PDAC and other PNI‐associated malignancies.

## Conflicts of Interest

The authors declare no conflicts of interest.

## Data Availability

Data sharing not applicable to this article as no datasets were generated or analyzed during the current study.

## References

[1] S. H. Chen , B. Y. Zhang , B. Zhou , C. Z. Zhu , L. Q. Sun , and Y. J. Feng , “Perineural Invasion of Cancer: A Complex Crosstalk Between Cells and Molecules in the Perineural Niche,” American Journal of Cancer Research 9, no. 1 (2019): 1–21. PMID: 30755808; PMCID: PMC6356921. 30755808 PMC6356921

[2] F. Nozzoli , M. Catalano , L. Messerini , et al., “Perineural Invasion Score System and Clinical Outcomes in Resected Pancreatic Cancer Patients,” Pancreatology 24, no. 4 (2024): 553–561, 10.1016/j.pan.2024.03.004. 38514359

[3] F. Liebl , I. E. Demir , K. Mayer , et al., “The Impact of Neural Invasion Severity in Gastrointestinal Malignancies: A Clinicopathological Study,” Annals of Surgery 260, no. 5 (2014): 900–907: discussion 907‐8, 10.1097/sla.0000000000000968. 25379860

[4] W. Tu , R. V. Gottumukkala , N. Schieda , L. Lavallée , B. A. Adam , and S. G. Silverman , “Perineural Invasion and Spread in Common Abdominopelvic Diseases: Imaging Diagnosis and Clinical Significance,” RadioGraphics 43, no. 7 (2023): e220148, 10.1148/rg.220148. 37319024

[5] J. Li , J. Ma , L. Han , et al., “Hyperglycemic Tumor Microenvironment Induces Perineural Invasion in Pancreatic Cancer,” Cancer Biology & Therapy 16, no. 6 (2015): 912–921, 10.1080/15384047.2015.1040952. 25946624 PMC4623034

[6] V. Anagnostopoulou , I. Pediaditakis , S. Alkahtani , et al., “Differential Effects of Dehydroepiandrosterone and Testosterone in Prostate and Colon Cancer Cell Apoptosis: The Role of Nerve Growth Factor (NGF) Receptors,” Endocrinology 154, no. 7 (2013): 2446–2456, 10.1210/en.2012-2249. 23696568

[7] G. O. Ceyhan , F. Bergmann , M. Kadihasanoglu , et al., “Pancreatic Neuropathy and Neuropathic Pain—A Comprehensive Pathomorphological Study of 546 Cases,” Gastroenterology 136, no. 1 (2009): 177–186e1, 10.1053/j.gastro.2008.09.029. 18992743

[8] F. Selvaggi , E. Melchiorre , I. Casari , et al., “Perineural Invasion in Pancreatic Ductal Adenocarcinoma: From Molecules Towards Drugs of Clinical Relevance,” Cancers (Basel) 14, no. 23 (2022): 5793, 10.3390/cancers14235793. 36497277 PMC9739544

[9] S. Schorn , A. Fritz , G. Kaissis , et al., “Neural Invasion Severity Is a Strong Predictor of Local Recurrence in Pancreatic Ductal Adenocarcinoma,” Surgery 180 (2025): 109018, 10.1016/j.surg.2024.109018. 39798180

[10] M. Schiavo Lena , G. Gasparini , S. Crippa , et al., “Quantification of Perineural Invasion in Pancreatic Ductal Adenocarcinoma: Proposal of a Severity Score System,” Virchows Archiv 483, no. 2 (2023): 225–235, 10.1007/s00428-023-03574-x. 37291275

[11] C. R. Ferrone , G. Marchegiani , T. S. Hong , et al., “Radiological and Surgical Implications of Neoadjuvant Treatment With FOLFIRINOX for Locally Advanced and Borderline Resectable Pancreatic Cancer,” Annals of Surgery 261, no. 1 (2015): 12–17, 10.1097/sla.0000000000000867. 25599322 PMC4349683

[12] C. L. Roland , A. D. Yang , M. H. G. Katz , et al., “Neoadjuvant Therapy Is Associated With a Reduced Lymph Node Ratio in Patients With Potentially Resectable Pancreatic Cancer,” Annals of Surgical Oncology 22, no. 4 (2015): 1168–1175, 10.1245/s10434-014-4192-6. 25352267 PMC5131370

[13] M. Mortoglou , D. Wallace , A. Buha Djordjevic , V. Djordjevic , E. D. Arisan , and P. Uysal‐Onganer , “MicroRNA‐Regulated Signaling Pathways: Potential Biomarkers for Pancreatic Ductal Adenocarcinoma,” Stresses 1, no. 1 (2021): 30–47, 10.3390/stresses1010004.

[14] M. Lian , M. Mortoglou , and P. Uysal‐Onganer , “Impact of Hypoxia‐Induced miR‐210 on Pancreatic Cancer,” Current Issues in Molecular Biology 45, no. 12 (2023): 9778–9792, 10.3390/cimb45120611. 38132457 PMC10742176

[15] S. S. Giri , A. S. Tripathi , P. Erkekoğlu , and M. E. A. Zaki , “Molecular Pathway of Pancreatic Cancer‐Associated Neuropathic Pain,” Journal of Biochemical and Molecular Toxicology 38, no. 4 (2024): e23638, 10.1002/jbt.23638. 38613466

[16] F. C. Wong , S. R. Merker , L. Bauer , et al., “Extracellular Vesicles From Pancreatic Cancer and Its Tumour Microenvironment Promote Increased Schwann Cell Migration,” British Journal of Cancer 132, no. 4 (2025): 326–339, 10.1038/s41416-024-02915-0. 39863771 PMC11832759

[17] Y. Sun , W. Jiang , X. Liao , and D. Wang , “Hallmarks of Perineural Invasion in Pancreatic Ductal Adenocarcinoma: New Biological Dimensions,” Frontiers Oncology 14 (2024): 1421067, 10.3389/fonc.2024.1421067. PMC11307098 39119085

[18] K. Murray , L. Oldfield , I. Stefanova , et al., “Biomarkers, Omics and Artificial Intelligence for Early Detection of Pancreatic Cancer,” Seminars in Cancer Biology 111 (2025): 76–88, 10.1016/j.semcancer.2025.02.009. 39986585

[19] X. Wang , R. Istvanffy , L. Ye , et al., “Phenotype Screens of Murine Pancreatic Cancer Identify a Tgf‐Alpha‐Ccl2‐Paxillin Axis Driving Human‐Like Neural Invasion,” Journal of Clinical Investigation 133, no. 21 (2023): e166333, 10.1172/jci166333. 37607005 PMC10617783

[20] T. S. Nagaria , H. Wang , D. Chatterjee , and H. Wang , “Pathology of Treated Pancreatic Ductal Adenocarcinoma and Its Clinical Implications,” Archives of Pathology & Laboratory Medicine 144, no. 7 (2020): 838–845, 10.5858/arpa.2019-0477-ra. 32023088 PMC9524086

[21] S. Borsekofsky , S. Tsuriel , R. R. Hagege , and D. Hershkovitz , “Perineural Invasion Detection in Pancreatic Ductal Adenocarcinoma Using Artificial Intelligence,” Scientific Reports 13, no. 1 (2023): 13628, 10.1038/s41598-023-40833-y. 37604973 PMC10442355

[22] R. Tang , Q. Meng , W. Wang , et al., “Head‐to‐Head Comparison Between FOLFIRINOX and Gemcitabine Plus Nab‐Paclitaxel in the Neoadjuvant Chemotherapy of Localized Pancreatic Cancer: A Systematic Review and Meta‐Analysis,” Gland Surgery 10, no. 5 (2021): 1564–1575, 10.21037/gs-21-16. 34164301 PMC8184396

[23] F. Wang , X. Xia , C. Yang , et al., “SMAD4 Gene Mutation Renders Pancreatic Cancer Resistance to Radiotherapy Through Promotion of Autophagy,” Clinical Cancer Research 24, no. 13 (2018): 3176–3185, 10.1158/1078-0432.ccr-17-3435. 29602802 PMC6345154

[24] I. E. Demir and C. Mota Reyes , “Chemokines: The (Un)usual Suspects in Pancreatic Cancer Neural Invasion,” Nature Reviews Gastroenterology & Hepatology 18, no. 4 (2021): 221–222, 10.1038/s41575-020-0329-1. 32561869

[25] Q. Xu , Z. Wang , X. Chen , et al., “Stromal‐Derived Factor‐1alpha/CXCL12‐CXCR4 Chemotactic Pathway Promotes Perineural Invasion in Pancreatic Cancer,” Oncotarget 6, no. 7 (2015): 4717–4732, 10.18632/oncotarget.3069. 25605248 PMC4467110

[26] H. Feng , K. Liu , X. Shen , et al., “Targeting Tumor Cell‐Derived CCL2 as a Strategy to Overcome Bevacizumab Resistance in ETV5(+) Colorectal Cancer,” Cell Death & Disease 11, no. 10 (2020): 916, 10.1038/s41419-020-03111-7. 33099574 PMC7585575

[27] F. Marchesi , L. Piemonti , G. Fedele , et al., “The Chemokine Receptor CX3CR1 Is Involved in the Neural Tropism and Malignant Behavior of Pancreatic Ductal Adenocarcinoma,” Cancer Research 68, no. 21 (2008): 9060–9069, 10.1158/0008-5472.can-08-1810. 18974152

[28] N. R. Jurcak , A. A. Rucki , S. Muth , et al., “Axon Guidance Molecules Promote Perineural Invasion and Metastasis of Orthotopic Pancreatic Tumors in Mice,” Gastroenterology 157, no. 3 (2019): 838–850e6, 10.1053/j.gastro.2019.05.065. 31163177 PMC6707836

[29] A. J. Rossi , T. M. Khan , H. Hong , G. B. Lesinski , C. Wu , and J. M. Hernandez , “Pepinemab (Anti‐sema4d) in Combination With Ipilimumab or Nivolumab for Patients With Resectable Pancreatic and Colorectal Cancer,” Annals of Surgical Oncology 28, no. 8 (2021): 4098–4099, 10.1245/s10434-021-10111-0. 33987757

[30] Q. Li , X. X. Zhang , L. P. Hu , et al., “Coadaptation Fostered by the SLIT2‐ROBO1 axis Facilitates Liver Metastasis of Pancreatic Ductal Adenocarcinoma,” Nature Communications 14, no. 1 (2023): 861, 10.1038/s41467-023-36521-0. PMC9932171 36792623

[31] A. Gohrig , K. M. Detjen , G. Hilfenhaus , et al., “Axon Guidance Factor SLIT2 Inhibits Neural Invasion and Metastasis in Pancreatic Cancer,” Cancer Research 74, no. 5 (2014): 1529–1540, 10.1158/0008-5472.can-13-1012. 24448236

[32] L. J. Martin , M. H. Piltonen , J. Gauthier , et al., “Differences in the Antinociceptive Effects and Binding Properties of Propranolol and Bupranolol Enantiomers,” Journal of Pain 16, no. 12 (2015): 1321–1333, 10.1016/j.jpain.2015.09.004. 26456674

[33] B. W. Renz , R. Takahashi , T. Tanaka , et al., “Beta2 Adrenergic‐Neurotrophin Feedforward Loop Promotes Pancreatic Cancer,” Cancer Cell 33, no. 1 (2018): 75–90e7, 10.1016/j.ccell.2017.11.007. 29249692 PMC5760435

[34] N. Wang , M. Yao , J. Xu , et al., “Autocrine Activation of CHRM3 Promotes Prostate Cancer Growth and Castration Resistance via CaM/CaMKK‐Mediated Phosphorylation of Akt,” Clinical Cancer Research 21, no. 20 (2015): 4676–4685, 10.1158/1078-0432.ccr-14-3163. 26071486

[35] S. M. Sousa , H. Branco , A. Avan , et al., “Darifenacin: A Promising Chitinase 3‐Like 1 Inhibitor to Tackle Drug Resistance in Pancreatic Ductal Adenocarcinoma,” Cancer Chemotherapy and Pharmacology 94, no. 4 (2024): 585–597, 10.1007/s00280-024-04712-1. 39225813 PMC11438711

[36] A. Szallasi , “Targeting TRPV1 for Cancer Pain Relief: Can it Work?,” Cancers (Basel) 16, no. 3 (2024): 648, 10.3390/cancers16030648. 38339399 PMC11154559

[37] E. Caronna , A. Alpuente , M. Torres‐Ferrus , and P. Pozo‐Rosich , “CGRP Monoclonal Antibodies and CGRP Receptor Antagonists (Gepants) in Migraine Prevention,” Handbook of Clinical Neurology 199 (2024): 107–124, 10.1016/B978-0-12-823357-3.00024-0. 38307640

[38] R. Covenas and M. Munoz , “Involvement of the Substance P/Neurokinin‐1 Receptor System in Cancer,” Cancers (Basel) 14, no. 14 (2022): 3539, 10.3390/cancers14143539. 35884599 PMC9317685

[39] I. Beirith , B. W. Renz , S. Mudusetti , et al., “Identification of the Neurokinin‐1 Receptor as Targetable Stratification Factor for Drug Repurposing in Pancreatic Cancer,” Cancers (Basel) 13, no. 11 (2021): 2703, 10.3390/cancers13112703. 34070805 PMC8198055

[40] I. Garajova and E. Giovannetti , “Targeting Perineural Invasion in Pancreatic Cancer,” Cancers (Basel) 16, no. 24 (2024): 4260, 10.3390/cancers16244260. 39766161 PMC11674953

[41] J. R. Ghilardi , K. T. Freeman , J. M. Jimenez‐Andrade , et al., “Administration of a Tropomyosin Receptor Kinase Inhibitor Attenuates Sarcoma‐Induced Nerve Sprouting, Neuroma Formation and Bone Cancer Pain,” Molecular Pain 6 (2010): 87, 10.1186/1744-8069-6-87. 21138586 PMC3004846

[42] M. Schmelz , P. Mantyh , A. M. Malfait , et al., “Nerve Growth Factor Antibody for the Treatment of Osteoarthritis Pain and Chronic Low‐Back Pain: Mechanism of Action in the Context of Efficacy and Safety,” Pain 160, no. 10 (2019): 2210–2220, 10.1097/j.pain.0000000000001625. 31145219 PMC6756297

[43] S. Jaffal and R. Khalil , “Targeting Nerve Growth Factor for Pain Relief: Pros and Cons,” Korean Journal of Pain 37, no. 4 (2024): 288–298, 10.3344/kjp.24235. 39322310 PMC11450303

[44] S. Na'ara , M. Amit , and Z. Gil , “L1CAM Induces Perineural Invasion of Pancreas Cancer Cells by Upregulation of Metalloproteinase Expression,” Oncogene 38, no. 4 (2019): 596–608, 10.1038/s41388-018-0458-y. 30171263

[45] D. D. Cave , M. Di Guida , V. Costa , et al., “TGF‐Beta1 Secreted by Pancreatic Stellate Cells Promotes Stemness and Tumourigenicity in Pancreatic Cancer Cells Through L1CAM Downregulation,” Oncogene 39, no. 21 (2020): 4271–4285, 10.1038/s41388-020-1289-1. 32291413 PMC7239770

[46] X. Xu , X. Lu , L. Chen , K. Peng , and F. Ji , “Downregulation of MMP1 Functions in Preventing Perineural Invasion of Pancreatic Cancer Through Blocking the NT‐3/TrkC Signaling Pathway,” Journal of Clinical Laboratory Analysis 36, no. 11 (2022): e24719, 10.1002/jcla.24719. 36181286 PMC9701873

[47] C. Hana , N. N. Thaw Dar , M. Galo Venegas , and M. Vulfovich , “Claudins in Cancer: A Current and Future Therapeutic Target,” International Journal of Molecular Sciences 25, no. 9 (2024): 4634, 10.3390/ijms25094634. 38731853 PMC11083183

[48] D. Tao , B. Guan , and C. Zhou , “Expression Patterns of Claudins in Cancer,” Heliyon 9, no. 11 (2023): e21338, 10.1016/j.heliyon.2023.e21338. 37954388 PMC10637965

[49] G. O. Ceyhan , I. E. Demir , M. Maak , and H. Friess , “Fate of Nerves in Chronic Pancreatitis: Neural Remodeling and Pancreatic Neuropathy,” Best Practice & Research Clinical Gastroenterology 24, no. 3 (2010): 311–322, 10.1016/j.bpg.2010.03.001. 20510831

[50] I. E. Demir , E. Tieftrunk , S. Schorn , et al., “Activated Schwann Cells in Pancreatic Cancer Are Linked to Analgesia via Suppression of Spinal Astroglia and Microglia,” Gut 65, no. 6 (2016): 1001–1014, 10.1136/gutjnl-2015-309784. 26762195

